# Supplementary material for: Understanding and predicting ciprofloxacin minimum inhibitory concentration in Escherichia coli with machine learning
Source: Sci Rep. 2020 Sep 14;10:15026. doi: 10.1038/s41598-020-71693-5 (PMC7490380; doi:10.1038/s41598-020-71693-5)
Supplement: Supplementary file 1 — Supplementary Information. [file 41598_2020_71693_MOESM1_ESM.pdf]

Understanding and predicting ciprofloxacin minimum inhibitory concentration in *Escherichia coli* with machine learning

Bálint Ármin Pataki[1,2,*], Sébastien Matamoros[3], Boas C.L. van der Putten[3,6], Daniel Remondini[4], Enrico Giampieri[5], Derya Aytan-Aktug[7], Rene S. Hendriksen[7], Ole Lund[8], István Csabai[1,2], Constance Schultsz[3,6], and COMPARE ML-AMR group[+]

[1]Department of Physics of Complex Systems, ELTE Eötvös Loránd University, Budapest, Hungary
[2]Department of Computational Sciences, Wigner Research Centre for Physics of the HAS, Budapest, Hungary
[3]Amsterdam UMC, University of Amsterdam, Department of Medical Microbiology, Amsterdam, The Netherlands
[4]Department of Physics and Astronomy (DIFA), University of Bologna, Bologna, Italy
[5]Department of Experimental, Diagnostic and Specialty Medicine (DIMES), University of Bologna, Bologna, Italy
[6]Amsterdam UMC, University of Amsterdam, Department of Global Health, Amsterdam Institute for Global Health and Development, Amsterdam, The Netherlands
[7]National Food Institute, Technical University of Denmark, Lyngby, Denmark
[8]Department of Bioinformatics, Technical University of Denmark, Lyngby, Denmark
[+]see the full list of the COMPARE ML-AMR group members in consortium section
[*]Corresponding author, email: patbaa@caesar.elte.hu

Supplementary materials

feature	Denmark	Italy	USA	UK	Vietnam	sum
gyrA#87	0.555	0.567	0.575	0.558	0.011	2.265
gyrA#83	0.115	0.148	0.114	0.141	0.690	1.209
parC#80	0.181	0.151	0.195	0.172	0.001	0.700
qnrS1	0.053	0.051	0.054	0.044	0.000	0.202
blaCTX-M-55	0.005	0.004	0.004	0.011	0.000	0.023
CP009072.1_3517597	0.000	0.001	0.000	0.000	0.011	0.012
CP009072.1_3517591	0.001	0.001	0.000	0.000	0.009	0.011
CP009072.1_1734215	0.001	0.001	0.000	0.001	0.009	0.011
CP009072.1_3517573	0.000	0.001	0.000	0.000	0.007	0.008
CP009072.1_113480	0.000	0.000	0.000	0.000	0.005	0.006
CP009072.1_459777	0.001	0.002	0.000	0.002	0.000	0.005
CP009072.1_1205372	0.002	0.001	0.001	0.000	0.000	0.004
CP009072.1_3517581	0.000	0.001	0.000	0.000	0.003	0.004
CP009072.1_3519619	0.000	0.000	0.000	0.000	0.003	0.004
CP009072.1_1205334	0.001	0.001	0.001	0.000	0.002	0.003

Table S1. Feature importances of the fitted random forest models. Random forest model was fitted on the training data using leave-one-country-out validation. Each entry shows the feature importance for the given feature for the validation step when the isolates from the given country were not used to train the model. Sorted by the sum of the feature importances. The features are following the gene # amino acid position naming where possible. For the mutations where there were no genes associated, the naming is chromosome name _ position. For the features coming from ResFinder, the ResFinder resistance gene naming was kept.

prediction (mg/L)	gyrA#87	gyrA#83	parC#80	qnrS1
0.018	No	No	No	No
0.060	No	No	Yes	No
0.276	No	Yes	No	No
0.331	No	No	No	Yes
0.448	Yes	No	No	No
0.921	No	Yes	Yes	No
1.105	No	No	Yes	Yes
1.496	Yes	No	Yes	No
5.109	No	Yes	No	Yes
6.917	Yes	Yes	No	No
8.303	Yes	No	No	Yes
17.046	No	Yes	Yes	Yes
23.079	Yes	Yes	Yes	No
27.705	Yes	No	Yes	Yes
128.077	Yes	Yes	No	Yes
427.339	Yes	Yes	Yes	Yes

Table S2. Parameters of the fitted linear regression model. The interception is -5.805, and the parameters associated with gyrA#87, gyrA#83, parC#80, qnrS1 are 4.648, 3.947, 1.738 and 4.211. Prediction is calculated as 2 to the power of the sum of interception and the present mutation/genes. Due to the nature of the linear regression model, it can extrapolate to unrealistically high values. Predictions can be clipped to a reasonable maximum value (eg. 64 mg/L), for simplicity that step was omitted.

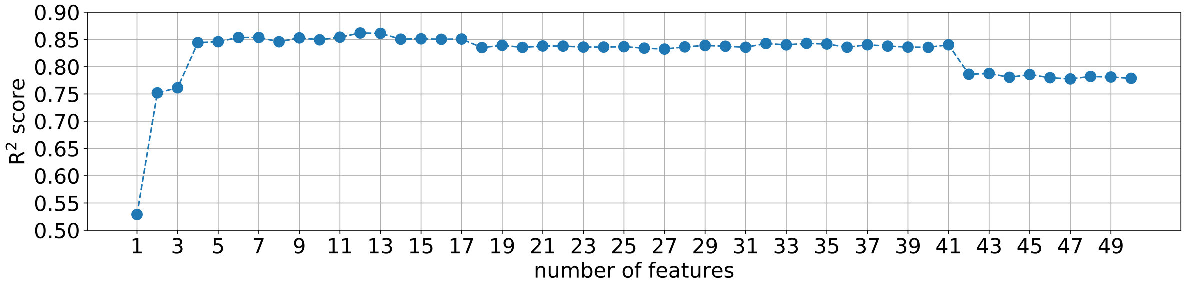

Figure S1. R squared score calculated on the training set using random forest model. The features were ranked based on Table S1 and iteratively a random forest model was fitted on the training set with leave-one-country-out validation using the top N features. The R squared score plateaued after N=4. The addition of more features did not improve the score significantly.

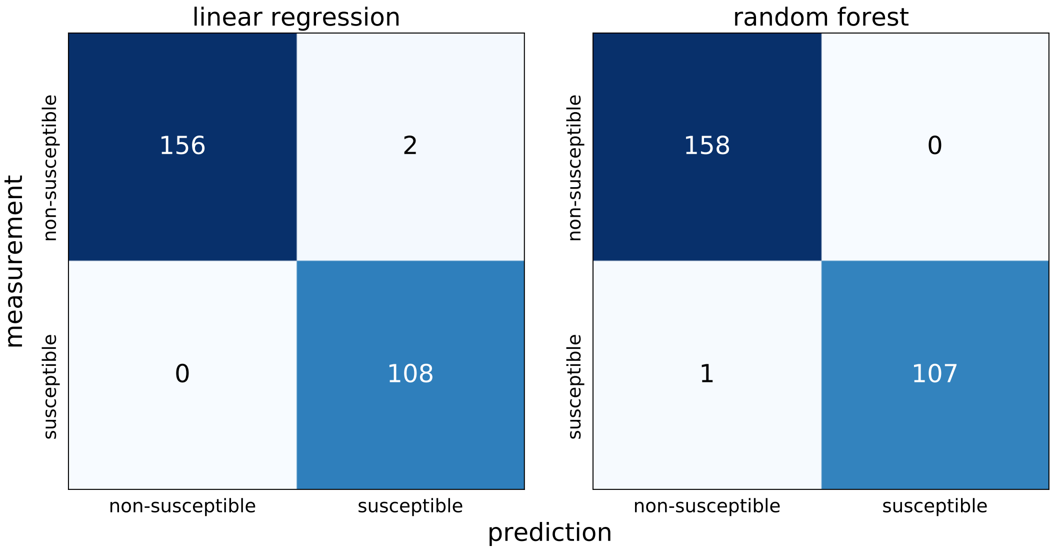

Figure S2. Confusion matrix for the linear regression and the random forest models. Both models were trained on the four selected features and were evaluated on the unseen test data. Binary outcomes were obtained via thresholding the MIC values. If the MIC value is higher than 1 mg/L, then the sample is considered to be non-susceptible, otherwise susceptible.

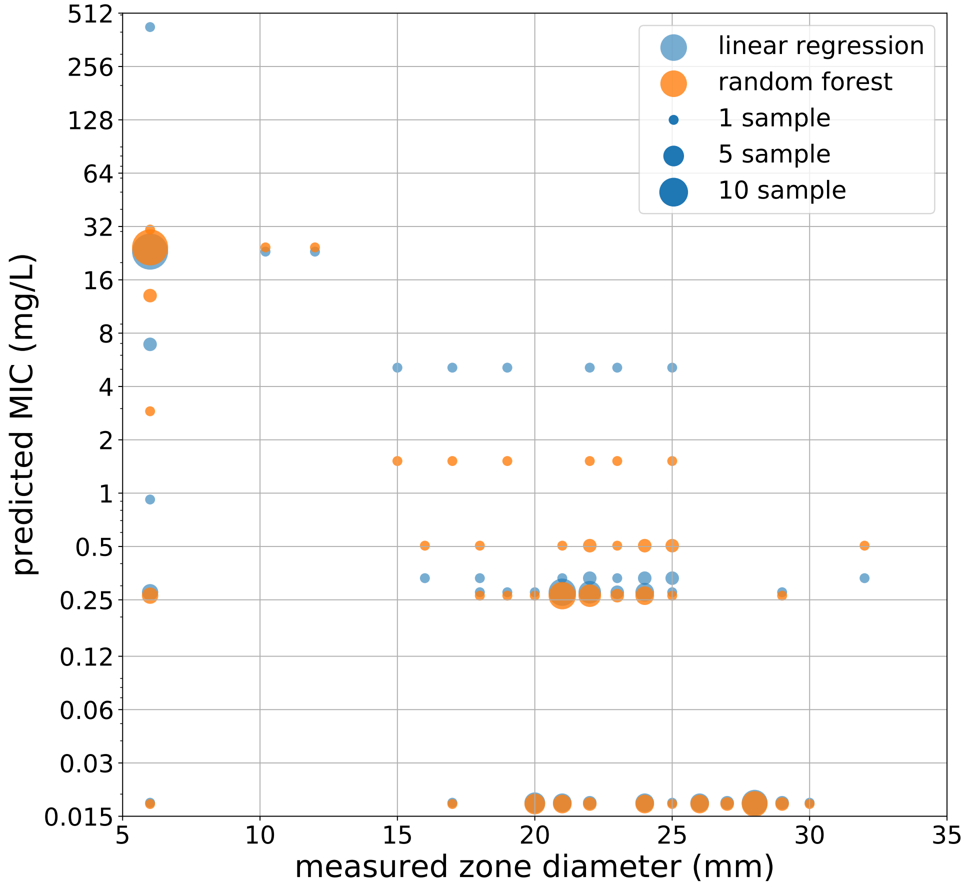

Figure S3. Prediction for samples that had only disk diffusion test measurement. As the larger zone diameter corresponds to smaller MIC values, a negative correlation is desirable on this plot. The same models were used with 4 predictors as it was used for the test set.

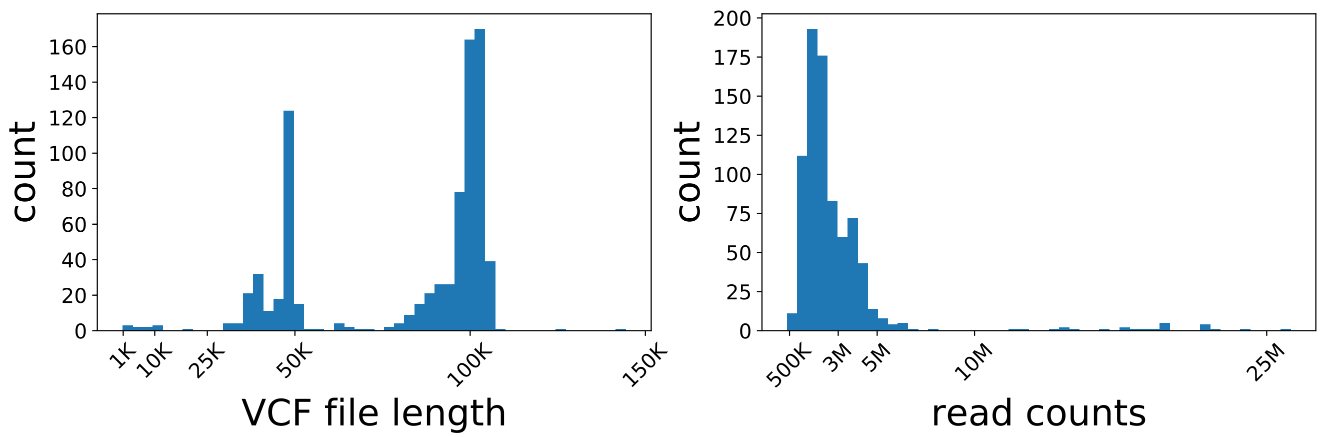

Figure S4. VCF file length distribution and the number of raw reads in the collected dataset.
